# Synthesis of indium nanoparticles at ambient temperature; simultaneous phase transfer and ripening

**DOI:** 10.1007/s11051-016-3683-8

**Published:** 2016-12-05

**Authors:** Mohammad Aghazadeh Meshgi, Manfred Kriechbaum, Subhajit Biswas, Justin D. Holmes, Christoph Marschner

**Affiliations:** 1 Institute of Inorganic Chemistry, Graz University of Technology, Stremayrgasse 9, Graz, Austria; 2 Materials Chemistry and Analysis Group, Department of Chemistry and the Tyndall National Institute, University College Cork, Cork, Ireland; 3 AMBER@CRANN, Trinity College Dublin, Dublin 2, Ireland

**Keywords:** Indium, Nanoparticle, Monodisperse, Colloidal solution, Reduction synthesis, SAXS

## Abstract

The synthesis of size-monodispersed indium nanoparticles via an innovative simultaneous phase transfer and ripening method is reported. The formation of nanoparticles occurs in a one-step process instead of well-known two-step phase transfer approaches. The synthesis involves the reduction of InCl_3_ with LiBH_4_ at ambient temperature and although the reduction occurs at room temperature, fine indium nanoparticles, with a mean diameter of 6.4 ± 0.4 nm, were obtained directly in non-polar *n*-dodecane. The direct synthesis of indium nanoparticles in *n*-dodecane facilitates their fast formation and enhances their size-monodispersity. In addition, the nanoparticles were highly stable for more than 2 months. The nanoparticles were characterised by dynamic light scattering (DLS), small angle X-ray scattering (SAXS), transmission electron microscopy (TEM), energy dispersive X-ray spectroscopy (EDS) and Fourier transform infrared (FT-IR) spectroscopy to determine their morphology, structure and phase purity.

## Introduction

Indium nanoparticles have been widely used as seeding particles for the growth of III-V semiconductor nanowires via solution or vapour-liquid-solid (VLS) mechanisms (Wang et al. 2006; Strupeit et al. 2009; Zhang et al. 2015b). In VLS growth, nanowire diameters and morphology can be controlled by the dimension of the catalyst (Barth et al. 2011). Due to the direct relationship between the diameter of nanoparticle seeds and the diameter and morphology of semiconductor nanowires grown from them, precise control over the diameter and morphology of nanoparticle seeds, such as indium, is important (Wang et al. 2006; Barth et al. 2011; Chockla et al. 2011; Guillemin et al. 2012). In addition to the synthesis of III-V semiconductor nanowires, ligand-free indium nanoparticles have also been successfully used as heterogeneous catalysts in the allylation of various carbonyl compounds (Dorn et al. 2016). Moreover, individual indium nanoparticles have been used for fabricating single-electron transistors which may be utilised in future nanometre-scale electronic devices (Junno et al. 1999).

Due to the diverse application of indium nanoparticles, their synthesis with different size distributions and morphologies has been reported using various methods, such as the reduction of indium salts with NaBH_4_ (Chou et al. 2008; Hammarberg and Feldmann 2009; Kind and Feldmann 2011; George et al. 2015), LiBH_4_ (Lim et al. 2010), sodium metal (Khanna et al. 2005) or alkalides and electrides (Tsai and Dye 1991). These reduction approaches at high temperatures result in a highly size-monodisperse spherical nanoparticles. In a two-step synthesis approach, the indium nanoparticles, initially synthesised by NaBH_4_ reduction of InCl_3_.4H_2_O in diethylene glycol at 100 °C, were transferred to a non-polar medium via oleylamine (particle diameters between 10 and 12 nm) (Hammarberg and Feldmann 2009). The synthesis of size-tunable (mean diameters between 8 and 105 nm), air stable, citrate-capped indium nanoparticles, via NaBH_4_ reduction of InCl_3_.4H_2_O in diethylene glycol at 100 °C is another example of high-temperature synthesis (Kind and Feldmann 2011). Furthermore, sub-10 nm spherical indium nanoparticles with uniform size and shape, capped with oleylamine, trioctylphosphine (TOP) or trioctylphosphine oxide (TOPO) have been synthesised by LiBH_4_ reduction of InCl_3_ under reflux conditions in isobutylamine (Lim et al. 2010). In all reported examples, uniform size-monodisperse nanoparticles were obtained by the reduction of an indium salt at high temperatures (Hammarberg and Feldmann 2009; Lim et al. 2010; Kind and Feldmann 2011). However, the synthesis of nanoparticles at elevated temperatures also increases the chance of nanoparticle oxidation (Vot et al. 2014; Dong et al. 2015). Another point associated with high-temperature synthesis is increased production cost at scale up (Masala and Seshadri 2004; Zhang et al. 2015a). In addition to the problems accompanying the synthesis of indium nanoparticles at high temperatures, due to the poor solubility of the indium salts in non-polar solvents, there is no report describing single-step synthesis of indium nanoparticles in non-polar solvents (Chou et al. 2008; Hammarberg and Feldmann 2009; Lim et al. 2010; Kind and Feldmann 2011; George et al. 2015). The only example of colloidal solutions of indium nanoparticles in non-polar solvents such as pentane or dodecane has been reported involving a two-step synthesis method, via the phase transfer of initially synthesised indium nanoparticles in a polar solvent with particle diameters between 10 and 12 nm. The report also lacks data on the degradation stability of the nanoparticles in non-polar solvents which is important for further applications (Hammarberg and Feldmann 2009). However, the initial formation of nanoparticles in a refluxing polar solvent followed by a time-consuming phase transfer process renders the two-step approach inefficient.

Herein, we report the direct, single-step formation of highly stable size-monodispersed indium nanoparticles with a mean particle size of 6.4 ± 0.4 nm in a non-polar medium through the reduction of anhydrous InCl_3_ with LiBH_4_ at room temperature using an innovative simultaneous phase transfer and ripening method. According to dynamic light scattering (DLS) analysis, the solution of indium nanoparticles does not show any aggregation even after 2 months. The described method should also be applicable to the direct, room temperature synthesis of other metallic nanoparticles in non-polar media without the requirement for a separate phase transfer step.

## Experimental

### Synthesis

All chemicals were used as received, except solvents which were either degassed under nitrogen for 1 h or put under vacuum. Synthesis of the indium nanoparticles was performed under dynamic nitrogen purging conditions. Indium nanoparticles were synthesised by the reduction of anhydrous InCl_3_ (ABCR, 98%) with a 2 M solution of LiBH_4_ in tetrahydrofuran (THF) (Sigma-Aldrich) in the presence of tri-*n*-butylphosphine (TBP) (Aldrich, 93.5%) as a capping ligand in *N*,*N*-dimethylformamide (DMF) (MERCK, 99%) as solvent at room temperature. Four hundred fifty microlitres (1.80 mmol) of TBP was added to 4.5 ml of a 40 mM solution of InCl_3_ (0.18 mmol) in DMF (in a Schlenk flask under dynamic nitrogen purging) and the solution mixture stirred for 15 min. Afterwards, 220 μl of a 2 M solution of LiBH_4_ (0.44 mmol) in THF was added dropwise over 90 s to the solution under vigorous stirring. Stirring was continued for 10 min after which 1 ml of the colourless solution was taken by syringe and injected very fast into each of the two non-polar systems and the whole mixture was stirred vigorously for 60 min. Non-polar system I contained 1.5 ml *n*-dodecane (Sigma-Aldrich, 99%) and 0.5 ml oleylamine (Aldrich, technical grade, 70%) while non-polar system II contained 1.5 ml *n*-dodecane (Sigma-Aldrich, 99%), 0.5-ml oleylamine (Aldrich, technical grade, 70%) and 56-mg (0.33 mmol) diphenylamine (DPA) (Sigma-Aldrich, 99%).

### Analytical tools

*Small angle X-ray scattering (SAXS)* measurement was performed with a high-flux SAXSess camera (Anton Paar, Graz, Austria) connected to a Debyeflex 3003 X-ray generator (GE-Electric, Germany), operating at 40 kV and 50 mA with a sealed-tube Cu anode. The Goebel-mirror focused and Kratky-slit collimated X-ray beam was line shaped (17 mm horizontal dimension at the sample) and scattered radiation from non-polar systems I and II, measured in the transmission mode was recorded by a one-dimensional MYTHEN-1k microstrip solid-state detector (Dectris Switzerland), within a q-range (with q being the magnitude of the scattering vector) of 0.1 to 5 nm^−1^. Using Cu Kα radiation of wavelength 0.154 nm and a sample-to-detector distance of 309 mm, this corresponds to a total 2θ region of 0.14 to 7°, applying the conversion q [nm^−1^] = 4π(sinθ)/*λ* with 2θ being the scattering angle with respect to the incident beam and *λ* the wavelength of the X-rays.

Samples were filled into a 1 mm (diameter) reusable quartz capillary with vacuum-tight sealing screw-caps at both ends. All measurements of the samples and blank were done with the capillary in vacuum and at 20°, with an exposure time of 10 min each. The scattering of the blank (*n*-dodecane) was subtracted from the scattering of the non-polar systems I and II (dispersed in *n*-dodecane, concentration = 0.2 mM) after normalising both spectra to the same transmission. Background-corrected scattering data of the samples were analysed by applying the indirect Fourier transformation method, which evaluates the particle distance distribution function p(r) in real space by using the program GIFT (Bergmann et al. 2000).

*DLS* was performed on a Zetasizer Nano ZS from Malvern Instrument equipped with a He-Ne laser. The scattered light was recorded at a backscattering angle of 173°. Control of the instrument parameters such as cuvette position, temperature, measurement time and repetitions as well as data acquisition and analysis was achieved using Zetasizer Software V.6.12. A fluorescence quartz cuvette of 10 × 10 × 48 mm size (Hellma Analytics) was used for measurement of colloidal solution of nanoparticles.

*Transmission electron microscopy (TEM) analysis* was conducted on a JEOL 2100 operated at 200 kV in bright-field condition for imaging. Two drops of the colloidal solution of indium nanoparticles were placed on the TEM grid under nitrogen purging and afterwards the grid was put under vacuum to remove any volatiles. The grid was kept under nitrogen inside a sealed vial and brought out of the vial just before analysis.

*Energy dispersive spectroscopy (EDS)* was carried out on FEI Helios NanoLab 600i operating at 20 kV and 0.69 nA equipped with Oxford X-Max 80 detector. The same TEM grid was used for EDS analysis.

*Fourier-transform infrared spectroscopy (FT-IR)* was performed on a Bruker Alpha FT-IR spectrometer equipped with Platinum ATR single reflection diamond. The spectra were acquired in a range between 3500 and 400 cm^−1^. To obtain the spectra of the nanoparticle-capped ligand, 2 ml of the colloidal solution of indium nanoparticles was mixed with 14 ml of DMF and 6 ml of THF and the mixture was stirred for 2 min followed by centrifugation at 2000 rpm for 5 min. Afterwards, the solution was removed and the precipitate was mixed with 10 ml of DMF and redispersed with ultrasonication. The solution mixture was then centrifuged for 6 min at 2000 rpm and afterwards the solution was removed and finally, the precipitate was put under vacuum for 10 min to remove any volatiles. The precipitate was directly subjected to FT-IR spectroscopy. The FT-IR spectra of the pure compounds were obtained by directly putting them onto the diamond cell.

*Nuclear magnetic resonance (NMR) spectroscopy* was performed on a Varian INOVA 300 (^31^P 121.4 MHz) spectrometer. A capillary filled with D_2_O was used for deuterium lock.

## Results and discussion

**Fig. 1:**
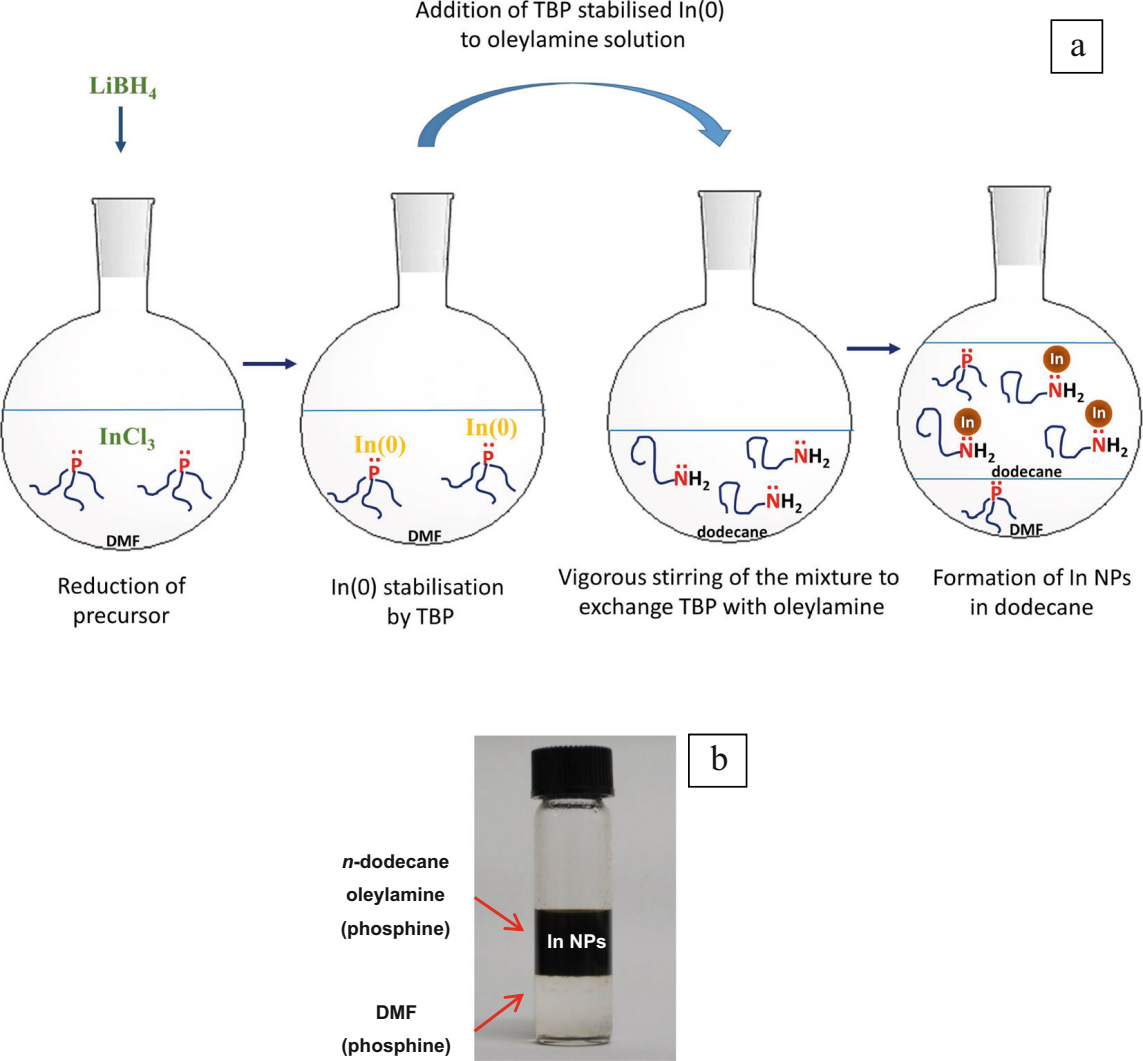
**a** Schematic representation of the In(0) formation and stabilisation by TBP. TBP exchange by oleylamine under vigorous stirring transfers In(0) species to the non-polar solvent and finally the accumulation of In(0) in the non-polar solvent and formation of indium nanoparticles. **b** Indium nanoparticles after phase transfer. The bottom transparent layer is DMF and the top layer is a mixture of *n*-dodecane, oleylamine and indium nanoparticles

Figure 1a illustrates schematically the procedure for the formation of indium nanoparticles according to the present study. Reduced InCl_3_ (using LiBH_4_ in DMF in the presence of TBP at ambient temperature) was mixed with oleylamine/*n*-dodecane under vigorous stirring, the colourless mixture turning to a dark brown/black solution. The mixture was then kept immobile to permit separation of the polar (DMF) from non-polar (oleylamine/*n*-dodecane) solvents containing the indium nanoparticles. TBP was distributed between both solvent phases due to its miscibility with both DMF and *n*-dodecane.

As the solubility of metal ion precursors in a non-polar medium is limited (Yang et al. 2011), InCl_3_ was dissolved in DMF as a polar solvent. After reduction of InCl_3_ with LiBH_4_ in DMF at room temperature, a stabiliser such as TBP is necessary to support the newly formed In(0) species in DMF and inhibit fast nucleation. The nucleation inhibiting behaviour of TBP can be observed from the colour change in the DMF solution. Colloidal solutions of sub-20 nm indium nanoparticles are typically dark brown in colour (Hammarberg and Feldmann 2009; Kind and Feldmann 2011). In contrast, the DMF solution containing TBP-stabilised In(0) species remains colourless for 1 h with a gradual colour change to pale yellow, which is a sign of In(0) nucleation. The solution turns a dark brown colour after stirring for 24 h. TBP plays a key factor in the stabilisation of In(0) species as discrete entities in DMF, acting as fast nucleation inhibitor. The absence of TBP results in the rapid nucleation of In(0) species and the formation of grey metallic indium particles. The slow nucleation of TBP-stabilised In(0) species and the very slow formation of indium nanoparticles in DMF, up to several hours, makes the transfer of discrete In(0) from DMF to a non-polar medium using a phase transfer reagent feasible. In this regard, before the nucleation of In(0) and the formation of indium nanoparticles in DMF, oleylamine transfers discrete In(0) species to a non-polar medium such as *n*-dodecane. In this case, vigorous stirring of the DMF solution containing In(0) mixed with oleylamine and *n*-dodecane promotes the transfer of discrete In(0) species from DMF to *n*-dodecane. In fact, instead of the previously reported phase transfer of nanoparticles from polar to non-polar medium (Hammarberg and Feldmann 2009), here, discrete TBP-stabilised In(0) species undergo the phase transfer reaction in the presence of oleylamine. Transfer of discrete In(0) to *n*-dodecane increases the concentration of In(0) species in *n*-dodecane which later promotes the nucleation and formation of indium nanoparticles in *n*-dodecane. In this regard, while the DMF solution of TBP-stabilised In(0) remains colourless for 1 h and turns to dark brown only after 24 h, the addition of the solution of DMF containing TBP-stabilised In(0) to a mixture of oleylamine/*n*-dodecane under vigorous stirring is accompanied by a rapid colour change from colourless to dark brown/black in less than 15 min. This rapid colour change in the presence of oleylamine confirms that the discrete TBP-stabilised In(0) species, after phase transfer to *n*-dodecane, undergo fast nucleation resulting in the rapid formation of indium nanoparticles in the mixture of oleylamine/*n*-dodecane.

**Fig. 2:**
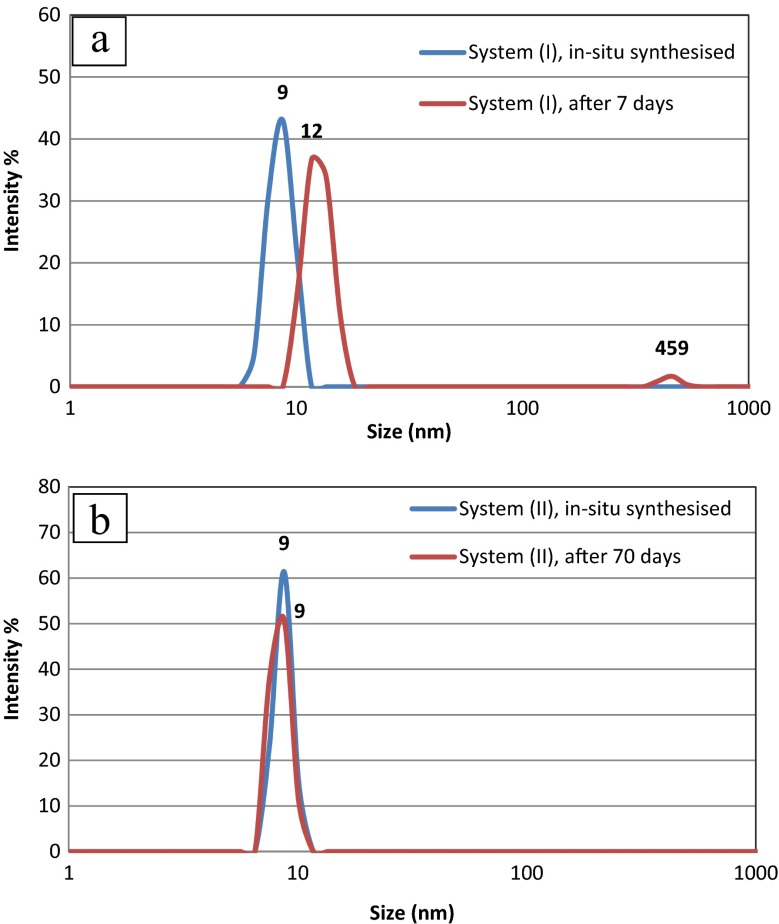
DLS intensity distributions of colloidal solution of indium nanoparticles. a Non-polar system I. *Blue line*: in situ synthesised sample. *Red line*: the same sample after 7 days. Although nanoparticles are size-monodispersed without any sign of agglomeration for a fresh sample, agglomeration is clearly visible in the aged sample. **b** Non-polar system II. *Blue line*: in situ synthesised sample. *Red line*: the same sample after 70 days. Nanoparticles are size-monodispersed without any sign of agglomeration in both the fresh and the aged samples

Separation of the polar and non-polar media was achieved by immobilising the mixture for a minimum time of 1 h; the non-polar medium forms the top layer, which due to the presence of indium nanoparticles is dark brown/black. During this process, the colourless DMF forms the bottom layer (Fig. 1b). Figure 2a shows DLS intensity distributions of the colloidal solutions of the indium nanoparticles in non-polar system I, i.e. only containing *n*-dodecane and oleylamine. The blue line represents the in situ prepared sample and the red line corresponds to the same sample after 7 days. DLS analysis of the in situ prepared sample showed that the indium nanoparticles had a mean diameter of 9 nm. However, the colloidal solution was not stable over time and aggregation of nanoparticles occurred over a period of 7 days, as seen from the red curve which shifts towards a larger diameter regime. Conversely, Fig. 2b shows the DLS intensity distribution of a colloidal solution of indium nanoparticles prepared using non-polar system II, i.e. with the additional presence of DPA along with *n*-dodecane and oleylamine. Again, the blue line represents the in situ prepared sample whereas the red line was obtained from the same sample aged after 70 days. The DLS traces of the in situ prepared samples were very similar for both non-polar systems I and II, with a mean diameter of 9 nm and no sign of agglomeration. However, despite the similarity of both in situ samples, over a large time interval, the nanoparticles synthesised through non-polar system I degraded and showed signs of aggregation, whereas the nanoparticles obtained through non-polar system II remained stable even after 70 days (Fig. 2b). The only difference between non-polar systems I and II was the presence of DPA, which significantly improves the stability of the nanoparticles against aggregation.

**Fig. 3:**
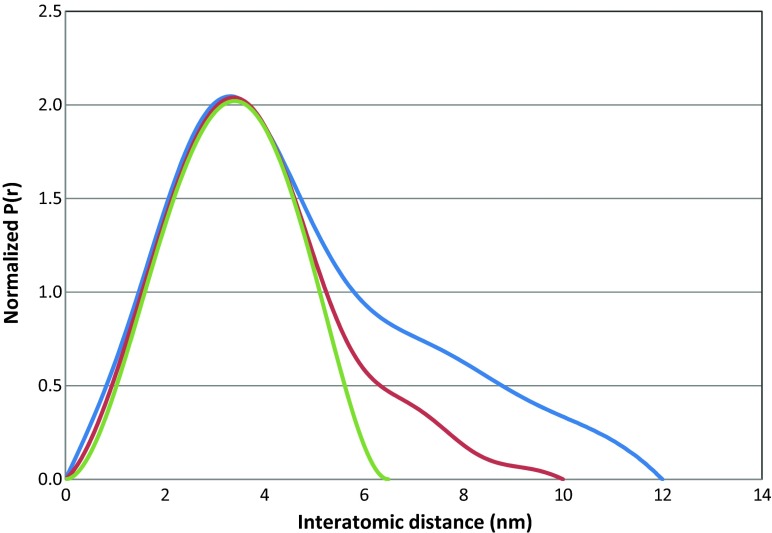
Distance distribution function p(r) of an in situ prepared colloidal solution of indium nanoparticles. *Blue line*: indium nanoparticles in the presence of oleylamine (system I). *Red line*: indium nanoparticles in the presence of oleylamine/DPA (system II). *Green line*: theoretically calculated distance distribution function of a size-monodispersed sample with symmetrical bell-shaped histogram. The system II sample with oleylamine/DPA has a narrower distance distribution and contains sub-10 nm particles, compared to sub-12 nm particles for system I

The small angle X-ray scattering (SAXS) distance distribution functions (related to the second moment of the histogram of distances within the particles) of in situ prepared colloidal solutions of indium nanoparticles obtained using systems I and II are depicted in Fig. 3. The blue line corresponds to indium nanoparticles with oleylamine (system I) whereas the red line represents indium nanoparticles with oleylamine/DPA (system II). The additional green line exemplifies a theoretically calculated distance distribution function of size-monodisperse spheres with symmetrical bell-shaped histogram and a mean diameter of 6.5 nm. Indium nanoparticles synthesised via both systems displayed a mean particle diameter of 6.5 nm (radius between 3.0 and 3.5 nm) but also contained contributions of larger particle sizes (10 and 12 nm, respectively). A comparison of the analytical data obtained from the SAXS and DLS techniques showed a difference of approximately 2 nm in the mean particle diameter. This difference in the mean particle diameter from both methods is due to the difference in the measurement techniques; SAXS data results from the electron density contrast between the sample and solvent (Schnablegger and Singh 2013) whereas DLS data are derived from the hydrodynamic diameter of the particles, which depends on the surface morphology of the particles (Lim et al. 2013) and any interactions between the particles and the medium (Kätzel 2007). Higher mean nanoparticle diameters from DLS measurements compared to SAXS analysis has previously been reported (Chen et al. 2012; Pabisch et al. 2012). However, DLS as a cheaper and quicker analysis technique is preferred over SAXS for the analysis of nanoparticles. According to the SAXS measurement shown in Fig. 3, the colloidal solution of indium nanoparticles synthesised via system I, with only oleylamine, exhibited a size distribution of particle diameters up to 12 nm, whereas the colloidal solution of indium nanoparticles synthesised via system II with oleylamine/DPA was up to 10 nm. The nanoparticle size distribution in the system I was broader than for nanoparticles synthesised with oleylamine/DPA (system II), displaying a narrower size distribution similar to a monodispersed symmetrical bell-shaped histogram. This data suggests that besides improving the long-term stability of the nanoparticles, the addition of DPA also has a positive effect in obtaining a homogeneously distributed indium nanoparticles.

**Fig. 4:**
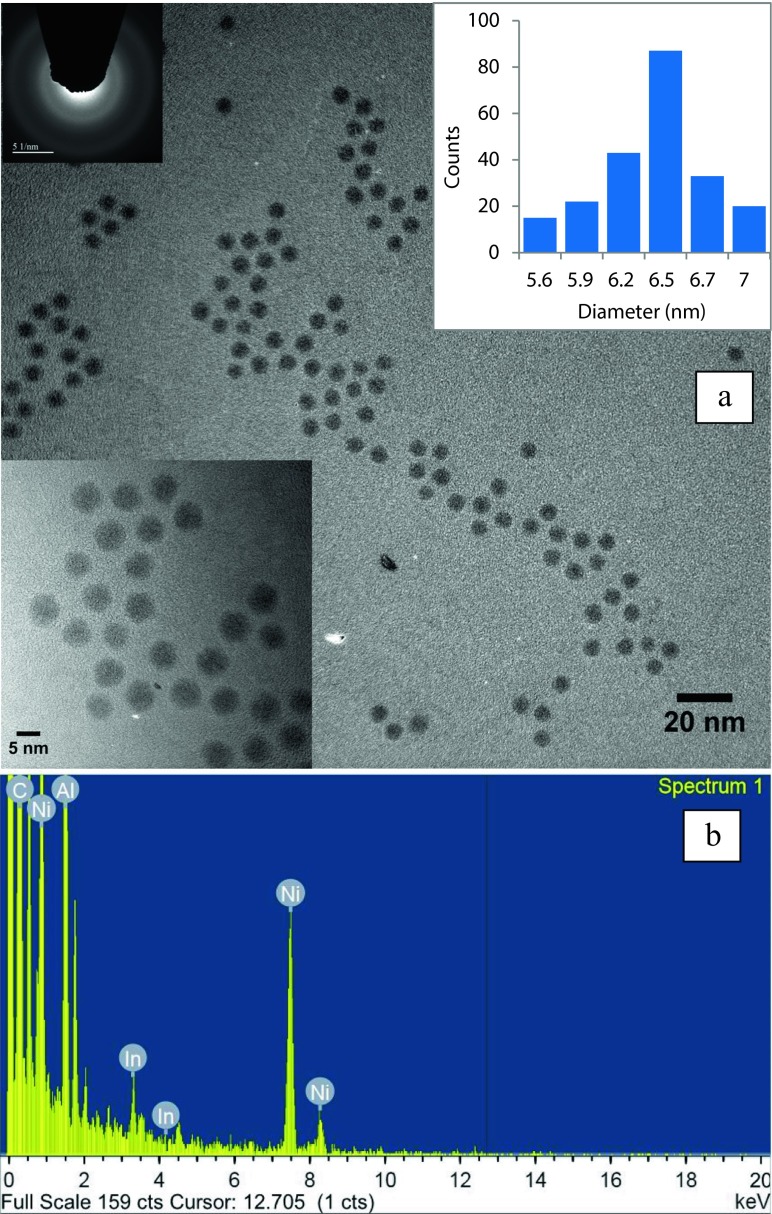
**a** Bright-field TEM image of indium nanoparticles synthesised in the presence of oleylamine/DPA (system II), with a mean diameter of 6.4 nm (Total number of nanoparticles = 220). *Inset* (*left-down*) shows nanoparticles in higher magnification, *inset* (*right-up*) shows histogram of the nanoparticles’ diameter with the standard deviation of 0.4 nm and *inset* (*left-up*) shows SAED pattern of amorphous nanoparticles. **b** EDS analysis of indium nanoparticles. Presence of Ni and Al and carbon relates to the TEM grid

Figure 4a shows a bright-field TEM image of the indium nanoparticles using the system II approach with the nanoparticles’ size histogram displaying a mean diameter of 6.4 nm and standard deviation of 0.4 nm (total number of nanoparticles analysed = 220), confirming the size-monodispersity of the sample. Large distances between individual nanoparticles indicated a well-dispersed and non-agglomerated colloidal solution. According to the TEM image, the mean particle size was 6.4 ± 0.4 nm, which was in accordance with the SAXS data. The higher diameter value obtained from the DLS data compared to SAXS and TEM analysis can be attributed to the effect of the hydrodynamic shell around the particles in solution (Pabisch et al. 2012).

The formation of small nanoparticles with a narrow diameter distribution at room temperature deserves special attention. It is known that the synthesis of nanoparticles at high temperatures increases the rate of nucleation and thereby decrease the particle size (Kwon and Hyeon 2011). The synthesis of indium nanoparticles via the reduction of indium salts has been previously reported at 100 °C resulting in the formation of nanoparticles with diameters between 10 and 12 nm (Hammarberg and Feldmann 2009). In another approach, indium nanoparticles were synthesised in reflux with mean diameters of 7.1 ± 0.5 nm, 7.8 ± 0.3 nm and 8.1 ± 1.1 by varying the capping ligand (Lim et al. 2010). In contrast, the synthetic procedure reported here allows for the formation of small nanoparticles with a narrow diameter distribution at room temperature, i.e. 6.4 ± 0.4 nm. The small mean particle diameter obtained in our approach, compared to nanoparticles synthesised by standard high-temperature methods, can be attributed to the saturation of DMF with TBP-stabilised In(0) species, where nucleation is inhibited by the stabilising effect of TBP. As soon as this solution is mixed with oleylamine/*n*-dodecane, an instantaneous colour change to yellow takes place which can be attributed to the formation of an infinite number of nuclei in a short moment, known as burst of nucleation (Kwon and Hyeon 2011). Burst of nucleation is related to the availability of a high concentration of In(0) species (or solute saturation) which instantaneously increases the chance of infinite nucleation and hence reduction of mean particle size. Another advantage of burst nucleation, according to classical LaMer nucleation and growth theory, is the formation of size-monodisperse colloidal solution of nanoparticles (LaMer and Dinegar 1950; LaMer 1952). Nucleation and growth of particles according to the LaMer mechanism is divided into three separate steps: (i) the formation of a high concentration of free atoms in solution; (ii) instantaneous nucleation of the free atoms or a nucleation burst, after which point no further nucleation is favourable due to the reduced concentration of the free atoms and (iii) growth of the initially formed nuclei. Due to the consumption of most of the free atoms during the second step, the concentration of the remaining free atoms is insufficient for the initiation of any new nucleation events and thus according to the third step, the remaining free atoms inside the solution will be consumed for the growth of the initially formed nuclei (LaMer and Dinegar 1950; LaMer 1952). In the synthesis of our nanoparticles, the nucleation burst drastically reduces the In(0) species in solution which hinders further nucleation. As a result the remaining In(0) will be consumed only for the growth of the initially formed nuclei. In general, the availability of the infinite TBP-stabilised In(0) species in DMF is the key factor for the nucleation burst when the DMF solution is mixed with oleylamine/*n*-dodecane and hence the formation of small nanoparticles with a narrow diameter distribution.

The relationship between the polarity of the solvent, or the capping ligand, and the morphology of nanocrystals and nanoparticles has been extensively studied (Sau and Murphy 2004; Khoza et al. 2012; Eastoe and Tabor 2014). The interaction between a solvent or capping ligand and the crystal interface determines the crystal growth rate in a preferential direction. High polarity solvents or capping ligands result in increased interaction with a particular interface inducing uniaxial growth (Sau and Murphy 2004; Xu et al. 2009; Xiao and Qi 2011). However, low polarity solvents or capping ligands promote isotropic and spherical growth of the crystals (Xu et al. 2009). As shown in Fig. 4a, the spherical shape of the indium nanoparticles produced in this study is due to the growth in a non-polar solvent, with minimum interaction between the solvent and the interfaces of the nanoparticles. Moreover, the growth of spherical nanoparticles highlights the isotropic interaction between the interface of the indium nanoparticles and oleylamine as the capping ligand.

Figure 4a (left-top inset) shows an SAED pattern of the nanoparticles with amorphous structure. The presence of amorphous nanoparticles is expected with room temperature synthesis and has been reported elsewhere (Soulantica et al. 2001). Figure 4b displays EDS analysis of indium nanoparticles showing the presence of characteristic indium peaks. The presence of Al, Ni and carbon in the EDS spectrum relates to the TEM grid.

**Fig. 5:**
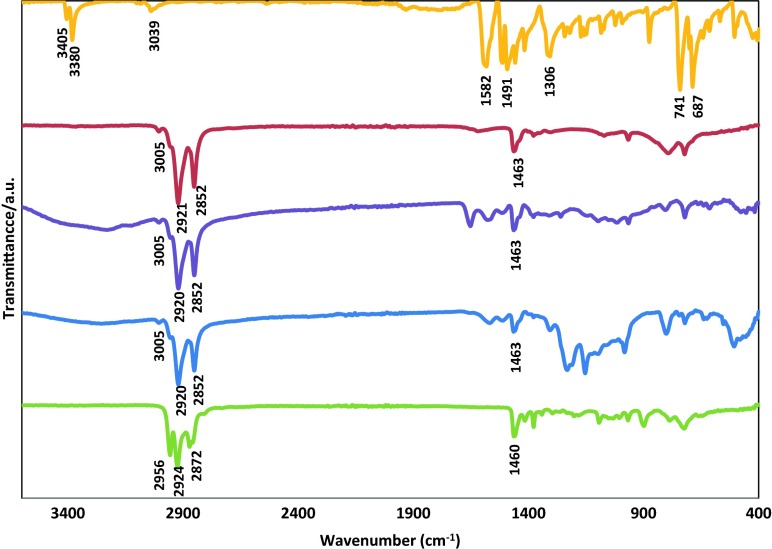
FT-IR spectra of pure DPA (*yellow line*), pure oleylamine (*red line*), indium nanoparticles synthesised from *n*-dodecane/oleylamine-DPA (system II) (*violet line*), indium nanoparticles from *n*-dodecane/oleylamine (system I) (*blue line*) and pure TBP (*green line*)

As evolution and ripening of indium nanoparticles occurs in a solution mixture containing TBP, oleylamine and DPA and as the ^31^P NMR spectroscopic analysis showed that TBP presented in both DMF and oleylamine/*n*-dodecane mixture after formation of the nanoparticles, it is important to determine what type of capping ligand or what mixture of them is attached to the indium nanoparticles. Figure 5 shows FT-IR spectra of indium nanoparticles synthesised in a mixture of oleylamine/*n*-dodecane/DPA (system II, violet line) and oleylamine/*n*-dodecane mixture (system I, blue line) and compared with pure oleylamine (red line) and pure TBP (green line). Characteristic IR vibrations of pure oleylamine at 3005, 2921, 2852, 1463 cm^−1^ were similar to both samples which were prepared with oleylamine and an oleylamine/DPA mixture. Although initially TBP stabilises In(0) in DMF, after the phase transfer reaction, TBP was not visible in the FT-IR spectrum, instead, oleylamine was present around indium nanoparticles.

For the non-polar system II, DPA is less basic than oleylamine due to the presence of electron withdrawing aromatic groups (Dewick 2013). Therefore, oleylamine is a better donor to indium nanoparticles than DPA. This can be confirmed by the absence of DPA in the FT-IR spectra of the indium nanoparticles (Fig. 5). Although DPA does not act as capping ligand, its presence in the solution mixture drastically improves the size-monodispersity and the long-term stability of the colloidal solution according to DLS results in Fig. 2b and TEM results in Fig. 4a. A credible role for stabilisation of nanoparticles with DPA can be its effect as a barrier between nanoparticles. To further study this effect, the amount of DPA was increased to 80 from 56 mg in an oleylamine/*n*-dodecane mixture (0.5 ml/1.5 ml). The solubility of 80 mg of DPA in solution was found to be the maximum solubility of DPA in the mentioned mixture. In this case, while the saturation of the solution mixture was achieved with DPA, precipitation of the indium nanoparticles out of the solution mixture was observed during the formation of indium nanoparticles. This precipitation of indium nanoparticles happened as the solution mixture was completely saturated with DPA thus providing no more space inside the solution for further accommodation of the bulky indium nanoparticles. This observation further suggests the role DPA plays as a barrier between nanoparticles.

## Conclusions

The synthesis of indium nanoparticles directly in a non-polar solvent and at room temperature was achieved using a new simultaneous phase transfer and ripening method, based on the phase transfer of single metallic atoms instead of bulky nanoparticles. Although the reduction of the indium precursor and the synthesis of nanoparticles occurred at room temperature, fine nanoparticles with the mean diameter of 6.4 ± 0.4 nm were obtained. The key factor for obtaining small diameter, size-monodisperse nanoparticles is due to the formation of TBP-stabilised In(0) species which trigger the burst nucleation and formation of fine nanoparticles. The addition of DPA decreased the distance distribution function of the nanoparticles, according to SAXS analysis, and improves the durability of the colloidal solution of nanoparticles to more than 2 months. This technique could be used for the synthesis of a broad range of metallic nanoparticles.
